# Prospective study of the 532 nm laser (KTP) versus diode laser 980 nm in the resection of hyperplastic lesions of the oral cavity

**DOI:** 10.4317/medoral.21812

**Published:** 2017-12-24

**Authors:** Patricia Bargiela-Pérez, Jorge González-Merchán, Rosa Díaz-Sánchez, Maria-Angeles Serrera-Figallo, Gerd Volland, Martin Joergens, Jose-Luis Gutiérrez-Pérez, Daniel Torres-Lagares

**Affiliations:** 1 DDS, PhD. Dental School. Universityof Seville, Spain; 2 DDS. Dental School. University of Seville, Spain; 3 DMD, PhD. Dental School. University of Seville, Spain

## Abstract

**Background:**

The aim of this study is to evaluate the resection of hyperplastic lesions on the buccal mucosa comparing the 532nm laser (KTP), versus diode 980nm laser, considering pain, scarring, inflammation and drug consumption that occurred postoperatively with each lasers.

**Material and Methods:**

A prospective study of consecutive series of 20 patients in two groups that presents hyperplastic lesions on the buccal mucosa. The choice of the KTP laser or diode 980nm laser for the surgery was made randomly. The power used was 1.5W in both groups in a continuous wave mode with a 320 μm optical fiber. Parameters of pain, scarring, inflammation and consumption of drugs were recorded by a Numerical Rating Scale and evaluated postoperatively. These recordings were made the day of the surgery, 24 hours after, 14 and 28 days after.

**Results:**

Pain and inflammation was light - moderate. The consumption of paracetamol was somewhat higher in the diode 980nm laser versus the KTP laser after 24 hours, although data was not statistically significant; significant differences were found after 28 days in regards to pain (*p* = 0.023) and inflammation (*p* = 0.023), but always in the absence parameter so we find no pain in both lasers. Scarring in the two types of laser showed no differences along the visits, with not detected scar retractable.

**Conclusions:**

Although there is a slight histological difference regarding the KTP laser in the oral soft tissues for clinical use, both wavelengths are very suitable for excision of oral fibroma.

** Key words:**Laser surgery, Laser therapy, oral surgery, soft tissue, 980 nm diode laser, 532 nm KTP laser.

## Introduction

Since Maiman in 1960 officially announced the use of the first laser (1), this technology became more and more popular. Currently laser can be found in many everyday applications. Lasers have been used in many professions, reaching the field of medicine, dentistry being no exception.

The use of laser therapy in the field of oral surgery is a common occurrence that has been developing in the last few years. Lasers in soft tissue surgery have been used since 1993, this technique acquired special importance in the field due to its many advantages in comparison to conventional surgery.

Laser technology has multiple applications in oral surgery, mainly used for the resection of soft tissue using thermal lasers such as diode, CO2 Argon or KTP. Removal of hard tissue is reserved for thermomechanical interacting Erbium wavelenghths such as ER:YAG or ER:YSGG .

In the field of laser technology, soft tissue surgery would be the application of choice. The different types of lasers give us the possibility, depending on the pathology that has to be treated, to choose the wavelength that is most beneficial for each injury.

The most common applications for soft tissue surgery are for: fibroms, mucoceles, frenectomies, vestibuloplastias, gingivectomies, angiomas, telangiectasias, leukoplakia, granulomas (2).

The oral mucosa is always susceptible to develop reactive and inflammatory reactions, because it is constantly subjected to both an external and internal stimuli.

The hyperplastic lesions represent the most frequently encountered oral mucosal lesions in humans. They may have a very different etiology and pathological features. Hyperplasia is the volume increase in tissue by the increase in cell numbers. Generally they correspond to a group of injuries caused by an exaggerated response of the oral mucosa versus low intensity chronic irritants. In the early stages, chronic irritant stimulates the formation of granulation tissue and endothelial proliferation; the tissue begins to undergo a process of fibroplasia. These lesions may regress early stages, but eventually tend to become fibrosed.

The most common surgical treatment of hyperplastic lesions of soft tissue in the oral mucosa is the complete excision of the lesion. Most hyperplastic lesions of the oral cavity correspond to benign tumors or soft tissue. In the case of the buccal mucosa, the most common injuries are: fibroma, lipoma and mucocele (3,4).

The first lasers used in soft tissue surgery were the Argon laser (488 nm), the CO2 (9600nm), the Nd: YAG laser (1064 nm) and diode laser (810nm / 980nm). Lasers with most studies in the surgical field are CO2, Nd: YAG, Er: YAG and diode (5,6).

Diode lasers have had a very important role in the field of soft tissue surgery in the last few years. Due to their high capacity of disinfection they are increasingly used for periapical surgery or periodontal treatment (7). These lasers are very effective in coagulation of superficial lesions, providing a dry surgical field (8). The light is constituted by a solid active medium, a semiconductor formed by using a combination of gallium, arsenic and other elements such as aluminum or indium for transforming electrical energy into light energy. They can be issued in either continuous or pulsed power and are easily transportable via optic fiber. The fiber has to be in contact with soft tissue for ablation procedures, incision and excision (9), because of the exit angle of about 42 degrees when the light exits the glass fiber. The KTP (532 nm) or “Green laser” is a solid laser with an active medium emitting in the visible portion of the spectrum (the beam is a very intense green light) produced by a diode (810 nm) laser acting as “pumps” of energy to stimulate a crystal of Nd: YAG (1064 nm). On the other hand, there is a potassium-titanyl-phosphate (KTiOPO4) crystal located between the gain medium and the semi-reflective mirror, this way the frequency is doubled meaning the reduction of the wavelength by half resulting in a beam emission of 532 nm (10).

This laser has been introduced in the field of medicine thanks to its high affinity for hemoglobin and oxyhemoglobin, becoming very effective in vascular lesions. Furthermore, in contrast to Nd: YAG, red coloured oral tissues absorb the light in the surface avoiding unpredictable deep tissue penetration and making the application much safer.

In Dentistry this laser was used for the first time with great success for bleaching (11). Romeo *et al.* ensure that the 532 nm dental laser is the most absorbed by hemoglobin, oxyhemoglobin and melanin given wavelength. This makes it a very suitable laser for vascular lesions absorbing only on the surface without penetrating into the surrounding tissue (10). There are not many studies on this laser in the field of oral surgery.

Fornaini *et al.* studied 52 patients affected with a benign pathology in the oral soft tissue with the 532 nm or KTP. They documented the intraoperative and postoperative pain with a numeric rating scale and the need of taking drugs. All cases were treated without anesthetic infiltration (Emdla® topical anesthesia was applied). They concluded that the use of this wavelength 532 nm at low power (1 W) and a fiber of 320 microns, provided a new tool in the treatment of benign soft tissue lesions in the oral cavity, with good healing, good ability to ablation and pain (12).

We found numerous studies on the application of diode laser resection of soft tissue and its benefits (9,13,14).

In recent years, there have been some studies on the use of the 532 nm or KTP wavelength in the excision of soft tissues with apparently promising results, however few scientific articles are written about it (10,15).

Since the use of the diode laser 980 nm was introduced a few years ago, surgical time has been greatly reduced for this type of pathology. Suturing is not necessary in most cases, and the use of painkillers has been greatly reduced. Although the initial penetration for oral tissue is higher, it is very quickly, limited by tissue alteration by dry out due to the water.

Contraindications found are primarily derived from improper use. Since the depth with which penetrate the surrounding tissue can cause thermal damage, misuse can affect improper postoperative scarring and less comfort for our patients.

In comparison the 532 nm laser is majorly absorbed on the surface and to a lesser extent in depth giving very promising results in the excision of soft tissue injuries (16,17).

Both lasers raised in this study use a 320-micron fiber system for transmission and same power settings resulting in the same power density and flow. Our question therefore was, what would be the most effective laser for the surgical use in the resection of benign hyperplastic lesions of the oral mucosa evaluating by a Numerical Rating Scale, the intra and postoperative pain, inflammation, scarring and drug consumption that occurred postoperatively in the two groups.

## Material and Methods

We present a prospective study of a consecutive series of 20 patients in two groups that present hyperplastic lesions on the buccal mucosa.

After having assessed whether or not the patients meet the inclusion criteria they were randomly assigned by a study coordinator who gave them identification numbers. These numbers were assigned a type of procedure. The number indicated whether the patient was to be operated with diode laser 980 nm or 532 nm laser, or KTP according to the protocol established.

The study population’s formed an initial sample of 24 patients of both sexes from the Department of Oral and Maxillofacial Surgery from The Hospital Universitario Virgen del Rocio in Seville, attending the laser unit of the Faculty of Dentistry of Seville.

These patients had already been previously diagnosed as having a hyperplastic lesion on the buccal mucosa, indicating excision as a treatment thereof. All persons gave their informed consent prior to their inclusion in the study. Of the initial sample, four patients were excluded for being smokers the more than 10 cigarettes a day (others exclusion criteria were diabetes mellitus, pregnant patients, patients treated with anticoagulants or patients treated with radiotherapy in the last 12 months).

The final sample consisted of 20 patients without history or disease of interest, of which 14 were women and 6 men, aged between 18 and 70 years.

All procedures performed in studies involving human participants were in accordance with the ethical standards of the institutional and/or national research committee and with the 1964 Helsinki declaration and its later amendments or comparable ethical standards.

For the study we used a diode laser from ARC MEDICAL LASER®, FOX model manufactured in Germany. It has a wavelength of 980 nm and a variable power of 1 to 10 W. The laser can work in continuous or discontinuous mode. It is a type IV laser according to American National Standards Institute (ANSI) classification.

The 532 nm laser, or KTP laser Green, also belonged to the ARC MEDICAL LASER® Classic Green Laser Model, Germany. It has a wavelength of 532 nm and a variable power of 1 to 12 Watts. It allows work both in pulsed or continuous mode. According to ANSI classification is a laser of high power type IV.

The optical fiber used is composed of silicate glass and has a thickness of 320 μm which is the most suitable for surgery. Disposable tips were used for each surgery, the active tip of the optical fiber should leave about 3 mm from the front of the handpiece and disposable plastic tip.

Both systems were programmed in the same power of 1.5 W in continuous wave mode, with 320 μm fiber, these parameters are recommended by the manufacturer and various authors used for the excision of this type of injury.

All photographic records were made with the same camera. Everything was recorded and performed by the same investigator including surgery, photographs and data collection.

Each patient had a notebook of data collection, in which all demographics were recorded and also the health conditions. For illustrative purposes we present one of the clinical cases operated for the study with the 532 nm laser.

**Figure 1:**
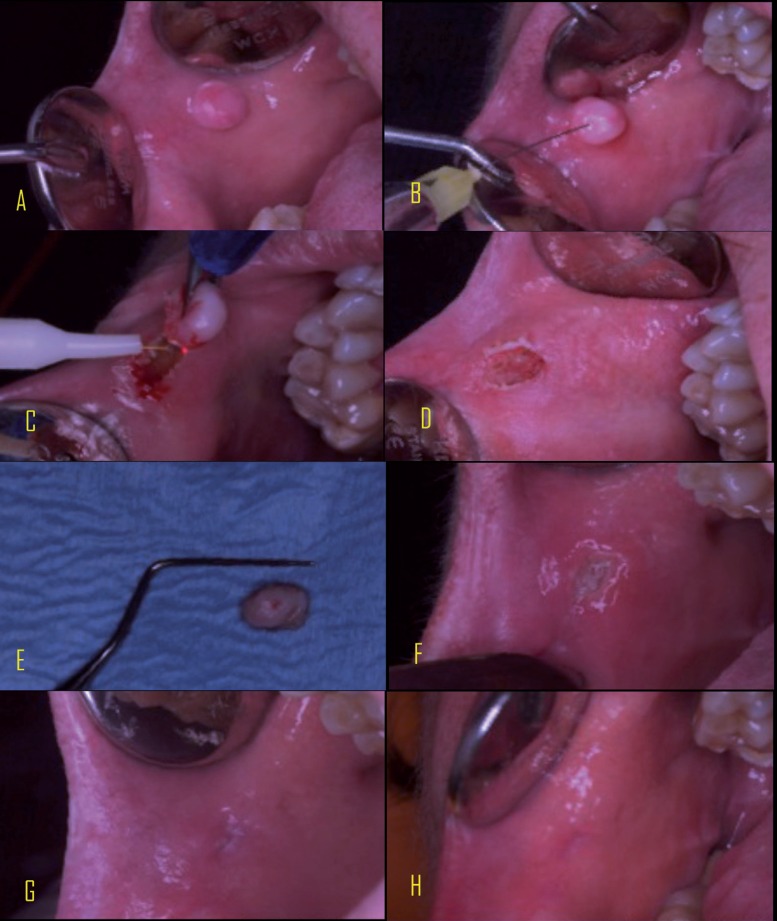
Diode laser group. A Initial view of lesion; B Anesthesia ; C Excision of the lesion; D Lesion excised; E Inmediate follow up; F24 h follow up; G 14 days follow up; H 28 days follow up.

**Figure 2:**
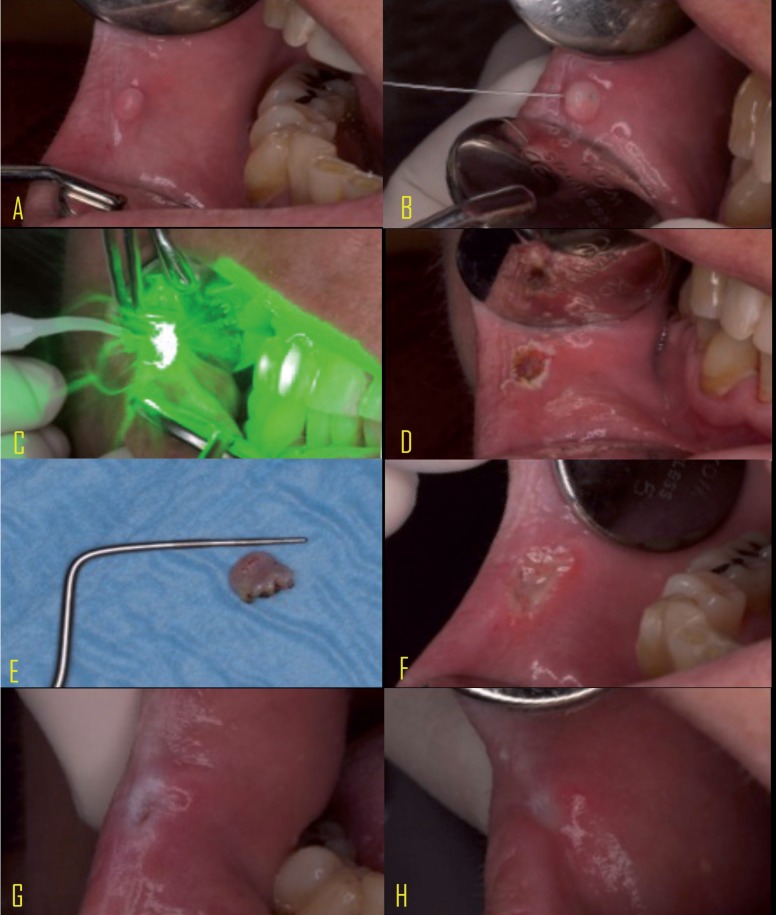
KTP laser group. A Initial view of lesion; B Anesthesia ; C Excision of the lesion; D Lesion excised; E Inmediate follow up; F24 h follow up; G 14 days follow up; H 28 days follow up.

To begin, an initial picture of the injury was taken at a distance of 20 cm (from camera to the patient) (Fig. 1A,2A).

The patient was further anesthetized with lidocaine and epinephrine hydrochloride (20 mg / ml + 0.0125 mg / ml) solution for injection, Xilonibsa INIBSA®, Spain, infiltrating only a quarter of carpule, reaching the line indicated in the Branded carpule 0.6 mg / ml (Fig. 1B,2B).

Once anesthetized adequate protection glasses were distributed among the staff and patient present in the room.

The excision of the lesion was performed using the selected laser. After removing the injury, the power to 0.5 W was changed and passed without contact over the injury for hemostasis and tissue biostimulation (Fig. 1C,D,2C,D).

After resection ended, a photograph was taken of the lesion with a periodontal probe to measure the size. The lesion was then sent to anatomical pathology (Fig. 1E,2E).

The postoperative care and recommendations to be followed were explained to the patient. The use of rinses were not allowed.

All patients had a Notebook Data Collection (NDC) which had to be filled and was verified by the investigator on the day of the intervention (Visit 0) in the appointment of 24 hours (Visit 1), 14 (Visit 2) and 28 days (Visit 3). The patient did a self assessment based on a visual analog scale, which measured the pain, the inflammation and scarring.

The pain felt by the patients was evaluated using a VAS scale of 1 to 5, where: 1 = no pain 2 = moderate pain 3 = pain 4 = severe pain 5 = very strong

The inflammation, mark on a VAS scale of 1 to 5 as follows: 1 = Absence of inflammation 2 = moderate inflammation 3 = Inflammation 4 = severe inflammation 5 = Very strong inflammation

The evolution of healing was picked up by the evaluator supported with the reason for the evaluation and photographs, where 1 = good, 2 = acceptable, 3 = poor healing. The clinical course of the soft tissues were recorded by intraoral pictures (Figs. 1F,G,H, 2F,G,H).

The intake of drugs was also recorded by the patient. They were prescribed only 1 gram Paracetamol only if they deemed necessary. If they took any medication prescribed or not, the time, the date and the amount had to be indicated in the NDC. Evaluation of postoperative parameters of pain, scarring, inflammation and consumption of drugs were recorded at the day of the surgery, 24 hours after, 14 and 28 days after. The researcher in the NDC had to fill out an evaluation form in an itinerary table.

Statistical analyses were performed using t-test chi-square, Brunner Langer, Mann-Whitney and Fisher Test of SPSS 20.0 (IBM, USA). The level of significance was predetermined as 0.05.

## Results

Twenty patients with an hyperplastic lesion in the oral mucosa were operated using a power of 1.5 W continuous wave in strict fiber contact. This data was collated from the literature review, in which the use of continuous waves for such resection indicates the use of lasers at low power (between 1 W and 2 W), whereas in the case of using a higher power (from 3 W) indications dictate it should be used pulsed mode or superpulsed to prevent thermal damage to surrounding tissues, especially with the use of the diode laser (18-20).

Regarding the 532 nm laser, the use of this laser for oral surgery is recommended to be used in low power 1 W to 1.5 W in continuous wave, obtaining very satisfactory results in surgeries (10,12,21). Studies of *in vitro* and *in vivo* use to determine an exact protocol for the resection of lesions with this type of soft tissue laser have not been developed.

The prevalence of bleeding and suture was the same in The distribution of time values with respect to the laser of 980 nm or 532 nm laser or KTP was similar in both groups (*p* = 0.436, Mann-Whitney).

-Postoperative pain:

On the study results, based on a strictly descriptive view, there is a greater proportion of subjects with mild to moderate pain using laser 980 nm versus 532 nm laser and KTP that is notorious that on the visit 1 (24 hours) . This difference in perception of pain may be related to the greater penetration into the tissue due to the lack of absorption surface of the diode laser 980 nm. At Visit 2 (14 days) they were no significant differences for pain between the two lasers. In the monthly visit (28 days), patients in both groups EVA declare a level of pain that is somewhat higher in the laser group or KTP 532 nm; but always classified in the category of ‘absence’ in any of the two groups, giving a statistically significant difference in the visit 3 (28 days) (*p* = 0.023) ( Table 1).

**Table 1:**
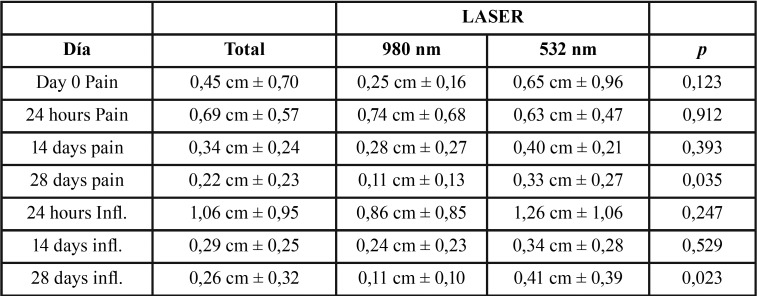
Results for Pain and Inflammation (Infl.).

-Inflammation:

Regarding inflammation, the data suggests that there is great consistency between the development of inflammation in both groups of patients. In the laser of 980 nm, a moderate inflammation occurs in some cases, but it is not statistically significant. Furthermore the Langer Brunner test result confirms that the evolution of inflammation is similar for both approaches (*p* = 0.989) ( Table 1).

Scarring:

Regarding healing in the two types of lasers along visits, no differences were detected between the two groups, and in both an acceptable healing. In none of the 20 cases there was infection ( Table 2).

**Table 2:**
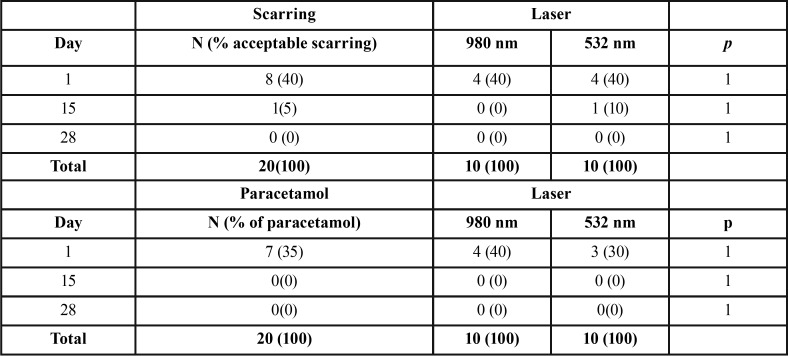
Results for scarring and drug consumption.

-Analgesic consumption:

By assessing the bar chart descriptively, it is observed that at Visit 1 (24 hours) in the laser diode of 980 nm, the consumption of Paracetamol is higher than in the 532 nm laser, but this does not provide statistically significant results. At visit 2 and visit 3, there was no consumption of 1 gram Paracetamol ( Table 2).

## Discussion

Previous studies have compared the laser with conventional surgery (16,22,23). However, there are no studies comparing the 980-nm diode laser and the 532 nm or KTP laser in oral hyperplastic lesions in the oral mucosa. In the current study pain peaking at 24 hours was observed in the 980 nm group descending from then on. For the rest of the review visits in both groups, the trend is the decline from the first time evaluated (1 day).

In our results, we note that the degree of pain on the VAS scale, the high value does not show any significant difference, so we can conclude that both lasers do not cause severe pain.

Several authors have described the absence of postoperative pain after surgery in the oral mucosa with various types of lasers (24-27).

In the monthly visit (28 days), patients in both groups EVA declare a level of pain that is somewhat higher in the 980 nm group or KTP 532 nm; but always is classified in the category of ‘absence’ in each groups.

So to be in this category of ‘absence’, the degree of pain can be admitted, in general, independent of the use of lasers of 980 nm or 532 nm laser, or KTP. This difference at 28 days, which always includes in part the absence of pain, may be due to measurements in VAS Scale, which are marked by the patient manually in the reviews.

In our study, none of the cases presented a retractable scar, in the reviewing at Visit 3 (28 days), as in the works of many authors, the absence of scar is recorded (9,26,28).

Considering the results obtained in our study, it is noteworthy that postoperative pain, (no-light on the VAS scale) without inflammation and with good healing occurs regardless of the laser used. Several authors agree that the postoperative course is very comfortable for patients because it is the result of less swelling and minimal inflammatory response (9,29-31).

This can be explained by the low laser damage produced by adjacent tissues, the lymphatic sealed and the formation of a fibrin clot over the wound to protect it from external irritations as we have shown above to give less pain and decreased risk of postoperative infection (12-18,32).

There are no clinical trials comparing these two types of oral soft tissue laser, however we found a histological study of Romeo *et al.* that compares these two wavelengths, and haves somewhat better results for the 532 nm laser and KTP. In the pathological examination both lasers showed a clearly defined zone of necrosis with no statistical difference in size. It confirms that the KTP laser produces less thermal damage depending on the tissue that is involved. Romeo *et al.* concluded that the difference between the two laser in the oral soft tissues was minimal (15).

This clinical trial had some limitations on such factors as pain measurement, being that it is subjective and influenced by numerous authors due to their culture and place of origin. Therefore VAS was employed, which can be understood by most patients and is reliable and can be reproduced (33-35). The measurement of drug consumption was also performed before the actual ingestion of the medication in order not to influence the results.

## Conclusions

The excision of benign hyperplastic tumors in buccal mucosa can be performed very safe and efficient with each laser, the 980 nm diode and the 532 nm KTP, used in the study. Although there is a little physical advantage in superficial absorption in the 532 nm for buccal mucosa the 980 nm works as well in clinically. After completed healing there are no differences visible or reported by patients.
